# Metagenomic Evidence for the Presence of Comammox *Nitrospira*-Like Bacteria in a Drinking Water System

**DOI:** 10.1128/mSphere.00054-15

**Published:** 2015-12-30

**Authors:** Ameet J. Pinto, Daniel N. Marcus, Umer Zeeshan Ijaz, Quyen Melina Bautista-de lose Santos, Gregory J. Dick, Lutgarde Raskin

**Affiliations:** a Infrastructure and Environment Research Division, School of Engineering, University of Glasgow, Glasgow, United Kingdom; b Department of Earth and Environmental Sciences, University of Michigan, Ann Arbor, Michigan, USA; c Department of Civil and Environmental Engineering, University of Michigan, Ann Arbor, Michigan, USA; University of British Columbia

**Keywords:** *Nitrospira*, comammox, drinking water systems

## Abstract

We report metagenomic evidence for the presence of a *Nitrospira*-like organism with the metabolic potential to perform the complete oxidation of ammonia to nitrate (i.e., it is a complete ammonia oxidizer [comammox]) in a drinking water system. This metagenome bin was discovered through shotgun DNA sequencing of samples from biologically active filters at the drinking water treatment plant in Ann Arbor, MI. Ribosomal proteins, 16S rRNA, and *nxrA* gene analyses confirmed that this genome is related to *Nitrospira*-like nitrite-oxidizing bacteria. The presence of the full suite of ammonia oxidation genes, including ammonia monooxygenase and hydroxylamine dehydrogenase, on a single ungapped scaffold within this metagenome bin suggests the presence of recently discovered comammox potential. Evaluations based on coverage and k-mer frequency distribution, use of two different genome-binning approaches, and nucleic acid and protein similarity analyses support the presence of this scaffold within the *Nitrospira* metagenome bin. The *amoA* gene found in this metagenome bin is divergent from those of canonical ammonia and methane oxidizers and clusters closely with the unusual *amoA* gene of comammox *Nitrospira*. This finding suggests that previously reported imbalances in abundances of nitrite- and ammonia-oxidizing bacteria/archaea may likely be explained by the capacity of *Nitrospira*-like organisms to completely oxidize ammonia. This finding might have significant implications for our understanding of microbially mediated nitrogen transformations in engineered and natural systems.

**IMPORTANCE** Nitrification plays an important role in regulating the concentrations of inorganic nitrogen species in a range of environments, from drinking water and wastewater treatment plants to the oceans. Until recently, aerobic nitrification was considered to be a two-step process involving ammonia-oxidizing bacteria or archaea and nitrite-oxidizing bacteria. This process requires close cooperation between these two functional guilds for complete conversion of ammonia to nitrate, without the accumulation of nitrite or other intermediates, such as nitrous oxide, a potent greenhouse gas. The discovery of a single organism with the potential to oxidize both ammonia and nitrite adds a new dimension to the current understanding of aerobic nitrification, while presenting opportunities to rethink nitrogen management in engineered systems.

## Abstract (precis)

Nitrification plays an important role in regulating the concentrations of inorganic nitrogen species in a range of environments, from drinking water and wastewater treatment plants to the oceans. Until recently, aerobic nitrification was considered to be a two-step process involving ammonia-oxidizing bacteria or archaea and nitrite-oxidizing bacteria. This process requires close cooperation between these two functional guilds for complete conversion of ammonia to nitrate, without the accumulation of nitrite or other intermediates, such as nitrous oxide, a potent greenhouse gas. The discovery of a single organism with the potential to oxidize both ammonia and nitrite adds a new dimension to the current understanding of aerobic nitrification, while presenting opportunities to rethink nitrogen management in engineered systems.

## OBSERVATION

Until recently, aerobic nitrification was considered to be a two-step process involving two functional guilds. The process of nitrification was considered split between ammonia-oxidizing bacteria (AOB) (1) and ammonia-oxidizing archaea (AOA) (2), which oxidize ammonia to nitrite, and strict nitrite-oxidizing bacteria (NOB) (3), which oxidize nitrite to nitrate. The phylogenetic distribution of AOB is limited to the *Betaproteobacteria* and *Gammaproteobacteria*; AOA fall within the *Thaumarchaea*, and NOB span the *Proteobacteria*, *Chloroflexi*, *Nitrospirae*, and *Nitrospinae*. However, the recent discovery of complete ammonia oxidizer (comammox) organisms, i.e., bacteria that completely oxidize ammonia to nitrate (4), within the genus *Nitrospira* has significantly changed our understanding of the aerobic nitrification process (5, 6). In the current study, we report metagenomic evidence of a *Nitrospira*-like organism that has the potential to perform both steps of the aerobic nitrification process and thus is likely to be a comammox bacterium. Specifically, it has genes to oxidize nitrite to nitrate (nitrite oxidoreductase) and possesses all genes required for ammonia oxidation, i.e., ammonia monooxygenase (*amoA*, *amoB*, *amoC*) and hydroxylamine dehydrogenase (also known as hydroxylamine oxidoreductase) (*hao*). This metagenome bin was discovered through shotgun DNA sequencing of samples from biologically active filters at a drinking water treatment plant (Ann Arbor, MI). IDBA-UD assembly (7), CONCOCT genome binning (8), and manual curation (see Text S1 in the supplemental material) resulted in 51 high-quality draft genomes with 2 to 755 scaffolds (median = 73), *N*_50_ ranging from 7.4 to 114 kbp (median = 86.9 kbp), and levels of completeness ranging from 77 to 100% (9). All genome bins were annotated by the Integrated Microbial Genomes Expert Review (IMG ER) system (10).

10.1128/mSphere.00054-15.1 Text S1  Additional descriptions of analyses. Download Text S1, DOCX file, 0.03 MB. Copyright © 2015 Pinto et al. 2015 Pinto et al. This content is distributed under the terms of the Creative Commons Attribution 4.0 International license.

**FIG 1:**
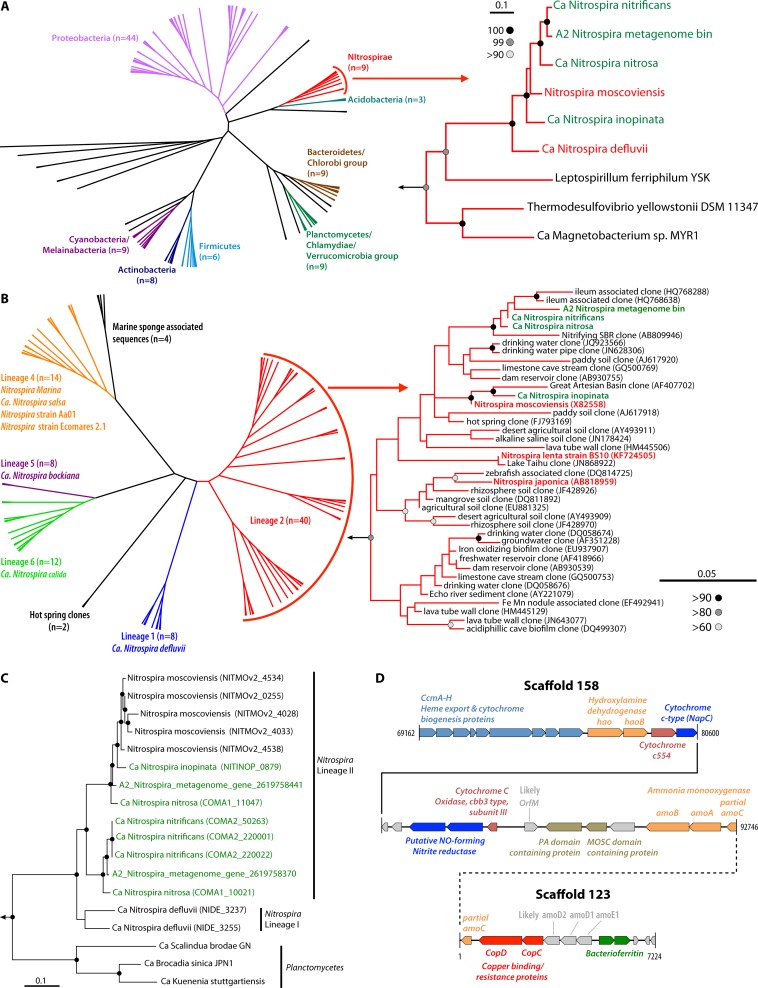
(A, left) Radial cladogram based on RAxML-based maximum-likelihood phylogeny (500 bootstraps, gamma distribution model, and LG+F substitution model) constructed using 16 syntenic ribosomal proteins, with prominent phylum-level affiliation of branches indicated. The reference sequence from the phylum *Aquificae* was used as the outgroup for this analysis. (Right) Expanded view showing the placement of the *Nitrospira* metagenome bin within the phylum *Nitrospirae*, with >90% bootstrap support indicated. The comammox *Nitrospira* species are in green, while strict NOB are in red. A detailed annotated tree is provided in the supplemental material, while the concatenated alignment used to perform phylogenetic analyses is available on figshare (http://dx.doi.org/10.6084/m9.figshare.1619897). (B, left) Radial cladogram based on RAxML-based maximum-likelihood phylogeny (1,000 bootstraps, gamma distribution model, GTR substitution model) constructed using 16S rRNA genes from 87 reference sequences within the genus *Nitrospira* and the partial 16S rRNA gene within the *Nitrospira* metagenome bin. The different lineages are in different colors. (Right) Expanded view of *Nitrospira* lineage 2, showing the placement of the 16S rRNA genome from the *Nitrospira* metagenome bin alongside recently published comammox *Nitrospira* organisms. Comammox *Nitrospira* bacteria are in green, while strict NOB are in red. (C) Bayesian inference phylogeny (20,000 generations, standard deviation = 0.02) *nxrA* genes from *Nitrospirae* and *Planctomycetes*, with the root placed on outgroup *Nitrococcus mobilis* (class *Gammaproteobacteria*)*.* Nodes with >99% bootstrap support are indicated with black circles. The *nxrA* genes from the *Nitrospira* metagenome bin cluster within lineage 2. (D) Arrangement of genes in the region from kbp 69.1 to 92.7 of scaffold 158 with ammonia oxidation genes and those on scaffold 123 with an arrangement similar to that of comammox *Nitrospira* bacteria. Hypothetical proteins are colored in gray, while genes annotated as coding for hypothetical proteins but showing homology to *orfM*, *amoD*, and *amoE* are also marked. The solid line indicates continuity between two fragments of scaffold 158, while the dotted line indicates likely connectivity between scaffold 123 and scaffold 158.

One of the genomes was phylogenetically assigned to the *Nitrospira* genus (JGI GOLD identification number Ga0074138) by Amphora2 (11). This 4.1-Mbp genome consists of 61 scaffolds (*N*_50_ = 150.74 kbp) with a GC content of 55%, 4,196 coding sequences, a complete 5S rRNA gene, and partial 16S and 23S rRNA genes. The genome bin was 88% complete, with 2.8% likely contamination based on 182 markers (9). The Amphora2 phylogenetic assignment was confirmed by Ribosomal Database Project (RDP) classification (12) of the 574-bp partial 16S rRNA gene in this genome (100% confidence). Maximum-likelihood phylogenetic analyses (13) of 16 syntenic ribosomal proteins (14) (Fig. 1A; see also Fig. S1 in the supplemental material) and the 16S rRNA gene (Fig. 1B) and Bayesian phylogenetic analyses (15) of the *nxrA* gene (Fig. 1C; Text S1) indicated that this metagenome bin is related to *Nitrospira* lineage II bacteria. The metagenome bin contains two copies of *nxrA*, both of which have high levels of similarity to *Nitrospira* lineage II bacterial genes (Fig. 1C). We were unable to recover the *nxrB* gene in the metagenome bin and suspect that this was due to assembly issues, as both copies of *nxrA* were found at the ends of their respective scaffolds. Indeed, mapping of the reads extracted for reassembly indicated that 391 paired-end reads mapped to the *nxrB* genes of organisms in *Nitrospira* lineage II, confirming that this gene was present but not assembled. As with *Nitrospira moscoviensis*, the new metagenome bin also contained a full suite of genes for urea transport and its degradation to ammonia (16).

10.1128/mSphere.00054-15.2 Figure S1  Detailed RAxML-based phylogenetic tree corresponding to the radial cladogram in Fig. 1A. The tree was constructed using a concatenated trimmed alignment of 16 syntenic ribosomal proteins (Text S1) using the gamma distribution model, LG+F substitution model, 500 bootstraps, and a fixed seed. The trimmed and concatenated alignment is available on figshare (http://dx.doi.org/10.6084/m9.figshare.1619897). Bootstrap support is shown at the nodes. Prominent phyla are colored corresponding to the radial cladogram in Fig. 1A. Download Figure S1, PDF file, 0.01 MB. Copyright © 2015 Pinto et al. 2015 Pinto et al. This content is distributed under the terms of the Creative Commons Attribution 4.0 International license.

The newly described metagenome bin contained genes involved in ammonia oxidation (*amoA*, *amoB*, *amoC*, and *hao*) on a single 92.7-kbp scaffold (scaffold 158; scaffold range, 101 to 161) in close succession on a region beginning at 69 kbp (Fig. 1D). This observation is consistent with reports of recently described comammox bacteria that have radically altered our understanding of the nitrogen cycle (5, 6) and provides further indication of the metabolic versatility of *Nitrospira* bacteria (16–18). In addition to finding the *amo* and *hao* genes, we found several genes on scaffold 158 and a second scaffold (scaffold 123) within this metagenome bin with a gene arrangement similar to that of the recently published comammox bacteria. First, the *hao* gene cluster was preceded by several genes associated with heme export and cytochrome *c* biogenesis (*ccmA* to *-H*), a feature not seen in any published AOB genomes and thus suggested as a diagnostic feature for comammox *Nitrospira* (5) (Fig. 1D). Second, we found a gene that likely codes for membrane-bound protein OrfM based on its similarity to the corresponding gene in “*Candidatus* Nitrospira inopinata” (identity = 69%, E value = 5e−132, bit score = 368) (5). However, IMG annotation and subsequent independent annotation efforts suggested that it was a hypothetical protein. Third, we found homologues of *amoD* (*n =* 2) and *amoE* arranged in close succession on scaffold 123, genes that were originally annotated as encoding hypothetical proteins. Their levels of identity to the corresponding genes in “*Ca.* Nitrospira inopinata” were 66% (E value = 1e−92) and 66.5% (E value = 4e−97) for *amoD* and 65% (E value = 2e−86) for *amoE*. These three genes were preceded by two genes annotated as encoding bacterioferritin and succeeded by the copper binding/resistance genes *copC* and *copD*, an arrangement consistent with that of the recently described comammox *Nitrospira* organism (5, 6). The *copD* gene was followed by a partial *amoC* gene at the 5′ end of scaffold 123, which is potentially contiguous to the partial *amoC* gene found at the 3′ end of scaffold 158. Despite these highly conserved features between our *Nitrospira* metagenome bin and the three other genome bins described recently, it is important to note that our observation is purely metagenomic in nature. Further experimental analyses need to be performed to (i) estimate the abundance of comammox *Nitrospira* organisms in the sampled drinking water filters and (ii) confirm the expression of these genes and link them to ammonia/nitrite oxidation.

**FIG 2:**
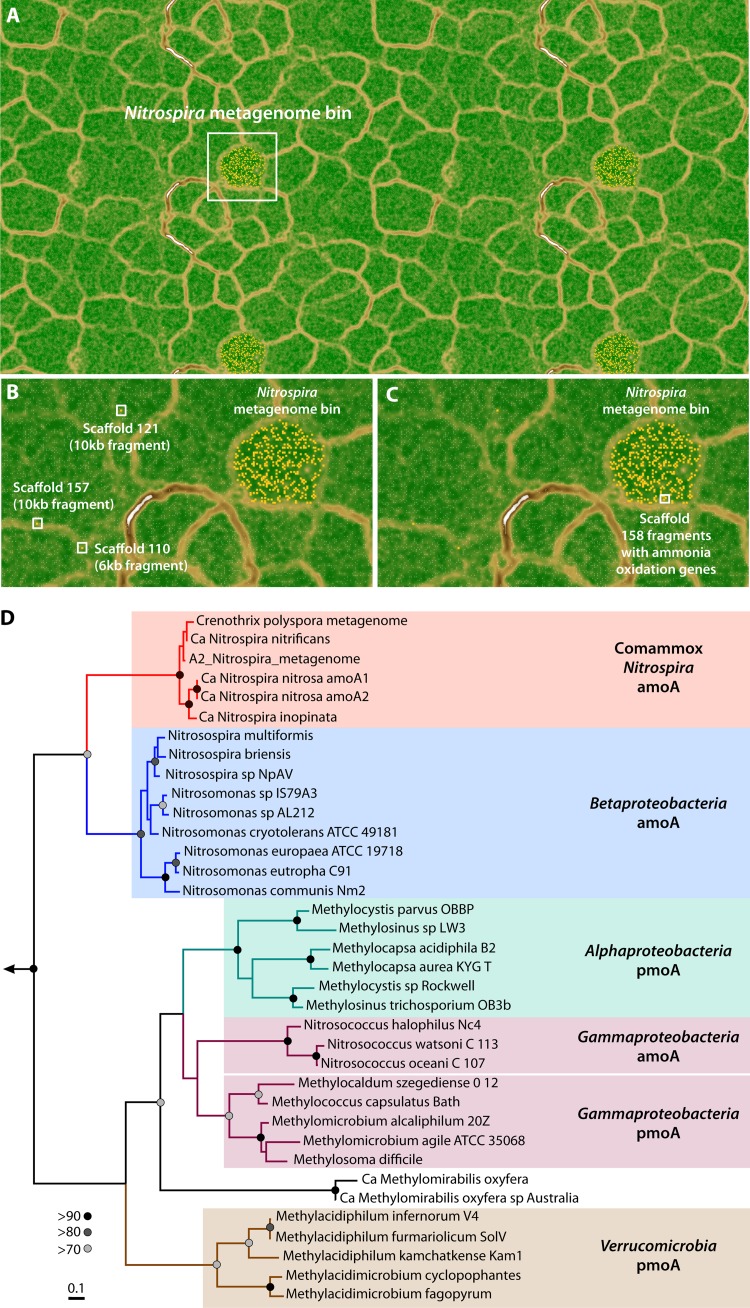
(A) Tiled view of an ESOM map constructed using all 51 metagenome bins assembled from the samples collected in this study, with the white square encompassing the *Nitrospira*-like metagenome bin. Some metagenome bins expand over the edge of a single ESOM grid. Hence, a tiled view consisting of four copies of the ESOM grid is shown to allow for visualization of metagenome bins at the edge as contiguous clusters. This results in all metagenome bins included in the ESOM analyses appearing four times in the tiled view. (B) Enlarged view of panel A indicating three scaffold fragments that were outliers based on ESOM analyses. (C) Enlarged view of panel A showing fragments of scaffold 158 containing ammonia oxidation genes that were binned with the *Nitrospira* metagenome. The ESOM binning procedure and contents of the three outlier scaffolds/scaffold fragments are presented in Text S1 in the supplemental material. (D) RAxML-based maximum-likelihood tree constructed using amino acid sequences of the *amoA* gene in the *Nitrospira* metagenome bin and *pmoA*/*amoA* sequences from a range of ammonia-oxidizing bacteria/archaea and methane-oxidizing bacteria, including the *Nitrospira* comammox. The tree was built from a trimmed muscle alignment using the Dayhoff model for protein evolution, gamma distribution model, and 500 bootstraps using the archaeal *amoA* gene of *Nitrosopumilus maritimus* as the outgroup. Branches are colored according to phylogenetic affiliation, and node support of >70% is indicated. This placement of the *amoA* gene from the *Nitrospira*-like genome and overall tree topology were also confirmed by neighbor-joining analysis (500 bootstraps) and the unweighted pair group method with arithmetic mean (UPGMA) (500 bootstraps) in Geneious and Bayesian phylogeny inference (20,000 generations) (Fig. S3).

To assess whether the presence of ammonia oxidation genes in the *Nitrospira*-like metagenome bin was an artifact, we considered the potential for (i) misannotation, (ii) misassembly, and (iii) incorrect genome binning. First, IMG ER annotation indicated strong support for *amoA*, *amoB*, and *hao* against sequences in the KEGG, InterPro, TIGR, and Pfam databases. *amoC* was confirmed only against the Pfam database (E value = 1.5e−25), potentially because it was a partial gene at the end of the scaffold. Second, we considered the likelihood of misassembly of scaffold 158 using coverage-per-base information (Fig. S2A and S2B) and also checked the phylogenetic signal along the length of the scaffold (Text S1). Our analyses confirmed that the scaffold not only was properly assembled (there is strong support for this conclusion from properly mapped reads) but also had regions of similarity to *Nitrospira*-like bacteria across nearly the entire scaffold length, including ribosomal protein L31P found on this scaffold, whose best hit was *Nitrospira* sp. ENR4 (NCBI accession number CUQ65102.1, total score = 132, query coverage = 100%, E value = 3e−38, percent identity = 88%), an enrichment of “*Ca.* Nitrospira inopinata.” Finally, we checked whether the scaffold was correctly binned into the *Nitrospira*-like metagenome bin. To do this, we compared the coverage per sample of scaffold 158 (Fig. S2C) and the k-mer frequency distribution (Fig. S2D) to those of other scaffolds in this metagenome bin. Both analyses suggested that this scaffold was not an outlier with respect to the other scaffolds in the bin. We also performed an Emergent Self-Organizing Map (ESOM) analysis (19) to test whether an independent binning approach would place the scaffold within this metagenome bin (Fig. 2A). ESOM analysis identified three outlier scaffolds unrelated to ammonia oxidation (Fig. 2B; Text S1), while the scaffold with ammonia oxidation genes binned with the *Nitrospira*-like metagenome bin (Fig. 2C). Based on these three lines of evidence (annotation, assembly, and binning), we conclude that the scaffold with ammonia oxidation genes belongs to the *Nitrospira*-like metagenome bin and thus suggests the presence of a comammox bacterium in the drinking water system of Ann Arbor, MI. The *amoA* gene of the newly reported *Nitrospira*-like metagenome bin clustered closely with the novel *amoA* gene of comammox *Nitrospira* bacteria. Phylogenetic analyses (15, 16) indicated that it branches from betaproteobacterial *amoA* but clusters closely with *pmoA* found in the metagenome of *Crenothrix polyspora* (Fig. 2C), a gammaproteobacterial methane oxidizer detected in a drinking water treatment plant in Germany (20), and “*Ca.* Nitrospira nitrificans” (6). It is interesting to note that one of the eight copies of 16S rRNA found in the *Crenothrix polyspora* metagenome on IMG belongs to a *Nitrospira*-like organism, suggesting that the gene annotated as *pmoA* might in fact be comammox *amoA*.

10.1128/mSphere.00054-15.3 Figure S2  Coverage per base for scaffold 158 within the *Nitrospira*-like genome using all reads used for reassembly (A) and properly mapped reads (both pairs mapped with correct insert sizes) (B). Two regions with higher-than-average coverage using properly mapped reads and the portion of the scaffold with ammonia oxidation genes are indicated. Per-sample coverage for all scaffolds (C) and comparison of k-mer frequency distributions for scaffold 158 and the median for all scaffolds (D) in the *Nitrospira*-like metagenome bin indicate that scaffold 158 is not an outlier. Download Figure S2, JPG file, 2.5 MB. Copyright © 2015 Pinto et al. 2015 Pinto et al. This content is distributed under the terms of the Creative Commons Attribution 4.0 International license.

10.1128/mSphere.00054-15.4 Figure S3  Bayesian inference phylogeny (20,000 generation, standard deviation = 0.017) for amino acid sequences of the *amoA* gene in the *Nitrospira* metagenome bin and *pmoA*/*amoA* sequences from a range of ammonia-oxidizing bacteria/archaea and methane-oxidizing bacteria, including a *Nitrospira* comammox. The root was placed on the archaeal *amoA* gene of *Nitrosopumilus maritimus*. Branches are colored according to phylogenetic affiliation, and nodes with >70% bootstrap support are indicated. Download Figure S3, PDF file, 0.1 MB. Copyright © 2015 Pinto et al. 2015 Pinto et al. This content is distributed under the terms of the Creative Commons Attribution 4.0 International license.

As part of this study, we also assembled a draft metagenome bin of *Nitrosomonas*-like AOB (GOLD identification number Ga0074132). However, due to the highly fragmented nature of this metagenome bin (598 scaffolds, *N*_50_ = 7.4 kbp), we were unable to recover all genes associated with nitrification. Nonetheless, its close affiliation with bacteria within the genus *Nitrosomonas* suggests that other canonical AOB were also present in these filters. To check for the presence of bacterial and archaeal *amoA* genes in the drinking water filter samples, we annotated the master assembly against a custom database of bacterial and archaeal *amoA* genes (Text S1). Only four significant *amoA* hits were detected in the master assembly, with two of these mapping to *Betaproteobacteria*-like *amoA*. The *amoA* gene within the *Nitrospira* metagenome bin was also detected in the master assembly, and we found an additional comammox-like *amoA* gene that was not present in any of the genomes assembled from this data set. Differences in coverage of the scaffolds on which these *amoA* genes were present across samples suggest that the four *amoA* genes (two comammox, two *Betaproteobacteria*) belong to four different organisms. The phylogenetic affiliations of all of these *amoA* genes and coverages per sample can be seen in Fig. S4.

10.1128/mSphere.00054-15.5 Figure S4  (A) RAxML-based maximum-likelihood phylogenetic analyses of *amoA* genes (*n =* 4) recovered in the master assembly along with reference *amoA*/*pmoA* genes as detailed in Text S1. Branches are colored according to phylogenetic affiliation nodes, and >70% bootstrap support is indicated. (B) Coverage of *amoA* genes detected in the master assembly per biosample. The top and bottom panels provide coverage information for *Nitrospira* and *Betaproteobacteria amoA* genes. Download Figure S4, PDF file, 0.1 MB. Copyright © 2015 Pinto et al. 2015 Pinto et al. This content is distributed under the terms of the Creative Commons Attribution 4.0 International license.

The presence of the complete ammonia oxidation capacity in *Nitrospira* organisms has significant implications for the nitrogen cycle, particularly if this organism is widespread. Using *amoA* sequence matches in the NCBI database and their associated environmental ontologies, we find support for previous detection of this *Nitrospira*-like organism in engineered systems (*n =* 8), soil ecosystems (*n =* 19), and groundwater (*n* = 10). This suggests that comammox *Nitrospira* organisms may be of importance for both engineered and natural systems. Interestingly, most of the matches in the NCBI database were attributed to methane-oxidizing bacteria, as also highlighted by Daims et al. (5). The recently published discovery of a comammox *Nitrospira* organism (5, 6) in combination with our finding strongly suggests that microbial contributions to the nitrogen cycle in engineered and natural environments will need to be reevaluated. The presence of a comammox is also congruent with previous observations of abundances of *Nitrospira*-like bacteria that were significantly higher than those of AOB/AOA based on 16S rRNA gene assays (21–23), indicating that comammox activity likely contributed substantially to nitrate formation in these environments. While direct evidence of the conversion of ammonia to nitrate by a *Nitrospira* organism was provided (5, 6), it will be critical to build on this initial work to understand the extent to which comammox organisms contribute to ammonia and nitrite oxidation in the wide range of environments where nitrogen cycling is important. This might be particularly critical for wastewater treatment systems that rely on partial nitrification followed by anammox processes (24) or short-cut nitrification-denitrification (25) for reducing energy costs of nitrogen removal. Similarly, nitrification in biofilms, a predicted ecological niche for comammox bacteria (4), is a considerable concern in drinking water distribution systems (26), and strategies devised to inhibit AOB/AOA may or may not yield optimal results if comammox activity primarily drives ammonia oxidation. On the other hand, the benefits of comammox bacteria can be exploited for ammonia removal from drinking water sources through promoting their activity in biofiltration systems, such as the system from which the currently described *Nitrospira*-like metagenome bin was obtained.

Raw reads and all draft genomes are available through NCBI BioProject PRJNA301005. The draft genomes and annotation information can be accessed through IMG ER using JGI GOLD identification numbers Ga0074129-141 and Ga0077522-560. JGI GOLD, IMG-ID, and NCBI accession numbers for the *Nitrospira* metagenome bin discussed in this paper are Ga0074138, 2619618852, and LNDU00000000, respectively.

## References

[1] Koops H-P, Purkhold U, Pommerening-Röser A, Timmermann G, Wagner M 2006 The lithoautotrophic ammonia-oxidizing bacteria, p 778–811. In Dworkin M, Falkow S, Rosenberg E, Schleifer K-H, Stackebrandt E (ed), The Prokaryotes. Springer, New York, NY. doi:10.1007/0-387-30745-1_36.

[2] Stahl DA, de la Torre JR 2012 Physiology and diversity of ammonia-oxidizing archaea. Annu Rev Microbiol 66:83–101. doi:10.1146/annurev-micro-092611-150128. 22994489

[3] Teske A, Alm E, Regan JM, Toze S, Rittmann BE, Stahl DA 1994 Evolutionary relationships among ammonia-oxidizing and nitrite-oxidizing bacteria. J Bacteriol 176:6623–6630. 7961414 10.1128/jb.176.21.6623-6630.1994 PMC197018

[4] Costa E, Pérez J, Kreft J-U 2006 Why is metabolic labour divided in nitrification? Trends Microbiol 14:213–219. doi:10.1016/j.tim.2006.03.006. 16621570

[5] Daims H, Lebedeva EV, Pjevac P, Han P, Herbold C, Albertsen M, Jehmlich N, Palatinszky M, Vierheilig J, Bulaev A, Kirkegaard RH, von Bergen M, Rattei T, Bendinger B, Nielsen PH, Wagner M 26 11 2015 Complete nitrification by *Nitrospira* bacteria. Nature doi:10.1038/nature16461. PMC5152751 26610024

[6] van Kessel MAHJ, Speth DR, Albertsen M, Nielsen PH, Op den Camp HJM, Kartal B, Jetten MSM, Lücker S 26 November 2015 Complete nitrification by a single microorganism. Nature doi:10.1038/nature16459. PMC4878690 26610025

[7] Peng Y, Leung HC, Yiu SM, Chin FY 2012 IDBA-UD: a de novo assembler for single-cell and metagenomic sequencing data with highly uneven depth. Bioinformatics 28:1420–1428. doi:10.1093/bioinformatics/bts174. 22495754

[8] Alneberg J, Bjarnason BS, de Bruijn I, Schirmer M, Quick J, Ijaz UZ, Lahti L, Loman NJ, Andersson AF, Quince C 2014 Binning metagenomic contigs by coverage and composition. Nat Methods 11:1144–1146. doi:10.1038/nmeth.3103. 25218180

[9] Parks DH, Imelfort M, Skennerton CT, Hugenholtz P, Tyson GW 2015 CheckM: assessing the quality of microbial genomes recovered from isolates, single cells, and metagenomes. Genome Res 25:1043–1055. doi:10.1101/gr.186072.114. 25977477 PMC4484387

[10] Markowitz VM, Chen I-A, Palaniappan K, Chu K, Szeto E, Grechkin Y, Ratner A, Jacob B, Huang J, Williams P, Huntemann M, Anderson I, Mavromatis K, Ivanova NN, Kyrpides NC 2012 IMG: the integrated microbial genomes database and comparative analysis system. Nucleic Acids Res 40:D115–D122. doi:10.1093/nar/gkr1044. 22194640 PMC3245086

[11] Wu M, Scott AJ 2012 Phylogenomic analysis of bacterial and archaeal sequences with AMPHORA2. Bioinformatics 28:1033–1034. doi:10.1093/bioinformatics/bts079. 22332237

[12] Wang Q, Garrity GM, Tiedje JM, Cole JR 2007 Naive Bayesian classifier for rapid assignment of rRNA sequences into the new bacterial taxonomy. Appl Environ Microbiol 73:5261–5267. doi:10.1128/AEM.00062-07. 17586664 PMC1950982

[13] Stamatakis A. 2014 RAxML version 8: a tool for phylogenetic analysis and post-analysis of large phylogenies. Bioinformatics 30:1312–1313. doi:10.1093/bioinformatics/btu033. 24451623 PMC3998144

[14] Castelle CJ, Hug LA, Wrighton KC, Thomas BC, Williams KH, Wu D, Tringe SG, Singer SW, Eisen JA, Banfield JF 2013 Extraordinary phylogenetic diversity and metabolic versatility in aquifer sediment. Nat Commun 4:2120. doi:10.1038/ncomms3120. 23979677 PMC3903129

[15] Ronquist F, Huelsenbeck JP 2003 MrBayes 3: Bayesian phylogenetic inference under mixed models. Bioinformatics 19:1572–1574. doi:10.1093/bioinformatics/btg180. 12912839

[16] Koch H, Lücker S, Albertsen M, Kitzinger K, Herbold C, Spieck E, Nielsen PH, Wagner M, Daims H 2015 Expanded metabolic versatility of ubiquitous nitrite-oxidizing bacteria from the genus *Nitrospira*. Proc Natl Acad Sci U S A 112:11371–11376. doi:10.1073/pnas.1506533112. 26305944 PMC4568715

[17] Palatinszky M, Herbold C, Jehmlich N, Pogoda M, Han P, von Bergen M, Lagkouvardos I, Karst SM, Galushko A, Koch H, Berry D, Daims H, Wagner M 2015 Cyanate as an energy source for nitrifiers. Nature 524:105–108. doi:10.1038/nature14856. 26222031 PMC4539577

[18] Koch H, Galushko A, Albertsen M, Schintlmeister A, Gruber-Dorninger C, Lücker S, Pelletier E, Le Paslier D, Spieck E, Richter A, Nielsen PH, Wagner M, Daims H 2014 Growth of nitrite-oxidizing bacteria by aerobic hydrogen oxidation. Science 345:1052–1054. doi:10.1126/science.1256985. 25170152

[19] Dick GJ, Andersson AF, Baker BJ, Simmons SL, Thomas BC, Yelton AP, Banfield JF 2009 Community-wide analysis of microbial genome sequence signatures. Genome Biol 10:R85. doi:10.1186/gb-2009-10-8-r85. 19698104 PMC2745766

[20] Stoecker K, Bendinger B, Schöning B, Nielsen PH, Nielsen JL, Baranyi C, Toenshoff ER, Daims H, Wagner M 2006 Cohn’s Crenothrix is a filamentous methane oxidizer with an unusual methane monooxygenase. Proc Natl Acad Sci U S A 103:2363–2367. doi:10.1073/pnas.0506361103. 16452171 PMC1413686

[21] LaPara TM, Hope Wilkinson K, Strait JM, Hozalski RM, Sadowksy MJ, Hamilton MJ 2015 The bacterial communities of full-scale, biologically active granular activated carbon biofilters are stable, diverse, and potentially contain novel ammonia-oxidizing microorganisms. Appl Environ Microbiol 81:6864–6872 doi:10.1128/aem.01692-15. 26209671 PMC4561712

[22] Dionisi HM, Layton AC, Harms G, Gregory IR, Robinson KG, Sayler GS 2002 Quantification of Nitrosomonas oligotropha-like ammonia-oxidizing bacteria and Nitrospira spp. from full-scale wastewater treatment plants by competitive PCR. Appl Environ Microbiol 68:245–253. doi:10.1128/AEM.68.1.245-253.2002. 11772633 PMC126567

[23] White CP, DeBry RW, Lytle DA 2012 Microbial survey of a full-scale, biologically active filter for treatment of drinking water. Appl Environ Microbiol 78:6390–6394. doi:10.1128/AEM.00308-12. 22752177 PMC3416602

[24] Kartal B, Kuenen JG, van Loosdrecht MCM 2010 Sewage treatment with anammox. Science 328:702–703. doi:10.1126/science.1185941. 20448175

[25] Peng Y, Zhu G 2006 Biological nitrogen removal with nitrification and denitrification via nitrite pathway. Appl Microbiol Biotechnol 73:15–26. doi:10.1007/s00253-006-0534-z. 17028876

[26] Zhang Y, Love N, Edwards M 2009 Nitrification in drinking water systems. Crit Rev Environ Sci Technol 39:153–208. doi:10.1080/10643380701631739.

