# Supplementary material for: Metagenomic Evidence for the Presence of Comammox Nitrospira-Like Bacteria in a Drinking Water System
Source: mSphere. 2015 Dec 30;1(1):e00054-15. doi: 10.1128/mSphere.00054-15 (PMC4863621; doi:10.1128/mSphere.00054-15)
Supplement: Text S1 [file sph001160049s5.docx]

**Supplemental Text S1**

**DNA sequencing, metagenomic assembly, genome binning and bin curation.**Samples were collected from the drinking water treatment plant in Ann Arbor, Michigan in December 2010, and February, May, and July 2011 and DNA was extracted as previously described (1). The extracted DNA was sequenced at the University of Michigan DNA sequencing core on two lanes of Illumina HiSeq 2000 resulting in a total of ~375 million 101 bp paired end reads. The reads were quality trimmed using sickle (2) using the sanger flag and trimmed reads from all samples were co-assembled using IDBA-UD version 1.1.1 (3) (minK =50, maxk=95, stepsize=5, pre_correction). This resulted in 223,135 scaffolds (135,158 scaffolds > 1 kbp) with an N_50_ of 14.3 kbp and maximum scaffold size of 979.2 kbp. After discarding scaffolds less than 2 kbp, the scaffold coverage per sample was estimated using a combination of bwa mem (4), samtools (5), and perl scripts (6). The scaffolds were clustered into metagenome bins using CONCOCT (7) and the resulting bins were evaluated for completeness based on single copy core genes (7). Genome bins with >80% completeness were manually curated to correct for potentially chimeric scaffolds whose fragments were assigned to more than one genome bin and to remove coverage and GC outliers. To refine the assemblies, reads mapping to each genome bin were extracted from all samples in the original dataset and each genome bin was independently reassembled using IDBA-UD (minK =20, maxk=100, stepsize=5, pre_correction) (3). Scaffolds less than 1 kbp were subsequently discarded and coverage per sample was calculated as described before. Reassembled genome bins were again assessed for completeness and manually curated to remove coverage and GC content outliers. Curated scaffolds were checked for completeness, contamination, and strain heterogeneity using CheckM (8).

**Phylogenetic analyses using 16 syntenic proteins, 16S rRNA, *nxrA* and *amoA* genes**.

We extracted 16 syntenic ribosomal protein sequences (9) from the *Nitrospira*-like metagenome bin, aligned each gene separately using a database of 116 diverse reference genomes (10) using muscle (11). The ribosomal proteins included in this analysis are rpL2, rpL3, rpL4, rpL5, rpL6, rpL14, rpL15, rpL16, rpL18, rpL22, rpL24, rpS3, rpS8, rpS10, rpS17, and rpS19. The reference genomes included 9 organisms from the phylum *Nitrospirae*, including two NOB (*Ca* Nitrospira defluvii*, Nitrospira moscoviensis*) and three comammox bacteria (*Ca* Nitrospira inopinata*, Ca* Nitrospira nitrificans*, and Ca* Nitrospira nitrosa). Each protein alignment was trimmed using trimal using the gappyout flag (12) and the best-fit model for protein evolution for each alignment was determined using prottest (13), which indicated the LG + F model (14) was the best fit for 15 of the 16 genes using both the Aikake and Bayesian information criteria. The trimmed alignments were concatenated into a 2,335 position superalignment and the best maximum likelihood tree was determined using RAxML (15) with 500 bootstraps and fixed seed. The resulting phylogenetic tree confirmed the Amphora2 and 16S rRNA gene classification results, placing the genome confidently within the *Nitrospirae* phylum where it clustered with *Ca* Nitrospira nitrificans with a bootstrap support of 100% (Figure 1A). For 16S rRNA based analyses, the partial 574 bp gene from the metagenome bin was aligned with 87 other 16S rRNA genes from genus *Nitrospira*, including 10 strict NOB and three comammox organisms. The alignment was performed in mothur (16) against the SILVA seed alignment (17). The alignments were trimmed using vertical =T and trump =. flags resulting in a 511 column trimmed alignment. RAxML (15) based maximum likelihood analyses was conducted with 1000 bootstraps with GAMMA model of rate heterogeneity and GTR substitution model. The *Nitrospira* assignment was also confirmed by Bayesian inference of phylogeny (18) using a MAFTT alignment (19) of *nxrA* genes from the genus *Nitrospira* and phylum *Planctomycetes* with the root placed on *nxrA* gene of *Nitrococcus mobilis* (Class: *Gammaproteobacteria*) (Figure 1B) (20,000 generations, standard deviation = 0.02). Reference *nxrA* genes were obtained from IMG or NCBI. RAxML based maximum likelihood was performed using amino acid sequences of the *amoA* gene in the *Nitrospira*-like metagenome bin and *pmoA*/*amoA* sequences from a range of ammonia oxidizing bacteria/archaea and methane oxidizing bacteria including genes from comammox *Nitrospira* genomes. The tree was built from a trimmed muscle alignment using the Dayhoff model for protein evolution, GAMMA model of rate heterogeneity and 500 bootstraps. The placement of the *amoA* gene from *Nitrospira*-like metagenome bin and overall tree topology was also confirmed by Neighbour-Joining (500 bootstraps) and UPGMA (500 bootstraps) in Geneious (http://www.geneious.com/), and Bayesian phylogeny inference (20,000 generations, standard deviation = 0.017) (Figure S3). Reference sequences obtained for all phylogenetic analyses were either obtained from NCBI, IMG, or from the publicly available genomes/genome assemblies of comammox bacteria.

**Evaluating scaffold 158 for misassembly.**

We considered the likelihood of scaffold misassembly to evaluate if genes from a different organism could have been merged with scaffold 158. The average coverage of the scaffold was ~41x, inclusive of all samples. An evaluation of the per-base coverage indicated a potential misassembly at position 33,357 with a maximum coverage of 215 at position 33,381 (Figure S2A). A closer inspection of this revealed that the five-fold higher coverage in this region was primarily due to incorrectly mapped reads (first read or mate pair unmapped or incorrect insert size). However, properly paired reads were found on either side of this region, supporting the assembly. We then filtered the sorted bam file (samtools view -f 2) to retain only those reads where both pairs were mapped to scaffold 158 with the correct insert size (~500 bp). This resulted in reduction in coverage around position 33,381 down to average scaffold coverage levels, while the overall coverage across the remaining portions of the scaffold remained largely unchanged, indicating that the scaffold was unlikely to be a result of misassembly (Figure S2B). Two regions still indicated 2x coverage compared to the scaffold average around the 19kb and 55kb. The first region coded for transposase and inactivated derivatives, while the second one coded for a putative efflux pump. However, we found significant support of paired reads extending both upstream and downstream of these regions into regions of scaffold with average coverage. Blastp analyses indicated that proteins in the high coverage regions and those immediately upstream and downstream consistently matched either *N. moscoviensis* or *Ca. N. defluvii* as their best hits. To further check for phylogenetic fidelity of scaffold assembly, we did a blastn search against the non-redundant NCBI database. Megablast found 11 alignments of this scaffold to the best hit organism *N. moscoviensis* ranging from position 580 to 33,472 (longest alignment = 3853, % identity = 76%, evalue = 0), while a discontinuous blastn analyses also indicated *N. moscoviensis* as the best hit with 41 aligned regions (longest alignment = 8940, % identity = 74, e value = 0) ranging from position 580 to position 87,492. Scaffold 158 also contained the ribosomal protein subunit L31P (co-ordinates: 1966-2172kb). Blastp analyses indicated best hits to *Nitrospira*-like organisms as shown below.

| **NCBI match** | **Total Score** | **Query Coverage (%)** | **E value** | **Percent identity** | **Accession number** |
| --- | --- | --- | --- | --- | --- |
| Nitrospira sp. ENR4 | 132 | 100 | 3e-38 | 88% | CUQ65102.1 |
| Nitrospira moscoviensis | 124 | 86 | 6e-35 | 95% | WP_053378624.1 |
| Nitrospira sp. 1 RAS filter enrichment | 124 | 86 | 8e-35 | 95% | CUS31554.1 |
| Nitrospira defluvii | 115 | 86 | 2e-31 | 86% | WP_013247564.1 |

**Emergent Self-Organizing Map analyses.**

For ESOM (20), we used all 51 metagenome bins assembled in this study and set a minimum scaffold size of 5 kbp, while cutting longer scaffolds into 10 kbp fragments (Figure 2A). Training was conducted using k-batch algorithm with 20 epochs. The number of rows and columns was set to 231 and 462, respectively. ESOM analyses marked three scaffolds binned into the *Nitrospira*-like metagenome bin by CONCOCT as outliers (Figure 2C). Specifically, scaffold 110 (6 kbp) and 10 kbp fragments at the 5’ end of scaffolds 121 and 157 were identified as outliers. A blastn analysis of scaffold 110 indicated 85% identity (evalue = 0, total score = 3949) to the best hit *Ca. N. defluvii*, while the 10 kb 5’ fragment of scaffold 121 coded for bacteriophage proteins (family HK97) that did not show any significant hit to sequences in NBCI’s non-redundant database. The 10 kb fragment of scaffold 157 coded for multiple hypothetical proteins and blastn analyses returned *Ca. N. defluvii* as its best hit (% identity: 71%, evalue = 0, query coverage = 96, total score = 4785). All remaining scaffolds were binned into the *Nitrospira*-like metagenome bin, including the 70-92.7 kbp regions (two fragments of scaffold 158), which contained the ammonia oxidation genes under consideration (Figure 1D).

***amoA* gene analyses in master assembly.**

To detect the presence of *amoA* genes in the entire dataset, we annotated the master assembly against a custom database of *amoA* genes. This included protein sequences from the pfam (archaea: pfam12942, number of sequences = 21965, bacteria: pfam02461, number of sequences = 24406) and the *amoA* genes from the *Nitrospira* metagenome bin from this study and from the three genomes published recently (21,22). Gene calling on the master assembly was done using prodigal (23) and the predicted protein were searched against the custom *amoA* database using rapsearch2 (25). This resulted in four significant hits shown below. All other matches either had an alignment length less than 60 positions and percent identity less than 50%.

| **Metagenome gene** | **Best hit** | **Percent identity** | **Alignment length** | **Log(e-value)** | **bitscore** |
| --- | --- | --- | --- | --- | --- |
| scaffold_574_2 | A2_Nitrospira_bin | 100 | 282 | -173.2 | 604.75 |
| scaffold_59415_2 | Ca_Nitrospira_inopinata | 88.6 | 281 | -155.35 | 545.43 |
| scaffold_78304_2 | Nitrosomonas sp. Strain Is79A3  pfam id: F8GI95_NITSI | 98.3 | 239 | -148.41 | 522.32 |
| scaffold_31055_5 | Uncultured prokaryote  pfam id: A9QV69_9BACT | 100 | 126 | -72.85 | 270.01 |

**SeqENv analyses**

The amoA gene sequences in the *Nitrospira*-line genome were used in SEQenv analyses (<http://environments.hcmr.gr/seqenv.html>). The SEQenv flags were set as follows: -t 2 –m 80 –q 40 –r 500 –s nucleotide. The *amoA* gene analyses resulted in 37 hits best matches. The SEQenv matches were than manually checked for environmental affiliation of the sequences.

**References.**

1. **Pinto AJ, Xi C, Raskin L.** 2012. Bacterial community structure in the drinking water microbiome is governed by filtration processes. Environ Sci Technol 46:8851-8859. doi: 10.1021/es302042t

2. **Joshi NA, Fass JN.** 2011. Sickle: A sliding-window, adaptive, quality-based trimming tool for FastQ files. https://github.com/najoshi/sickle.

3. **Peng Y, Leung HCM, Yiu SM, Chin FYL.** 2012. IDBA-UD: a de novo assembler for single-cell and metagenomic sequencing data with highly uneven depth. Bioinformatics 28:1420-1428. doi: 10.1093/bioinformatics/bts174

4. **Li H, Durbin R.** 2009. Fast and accurate short read alignment with Burrows–Wheeler transform. Bioinformatics 25:1754-1760. doi: 10.1093/bioinformatics/btp324

5. **Li H, Handsaker B, Wysoker A, Fennell T, Ruan J, Homer N, Marth G, Abecasis G, Durbin R, Subgroup GPDP**. 2009. The Sequence Alignment/Map format and SAMtools. Bioinformatics 25:2078-2079. doi: 10.1093/bioinformatics/btp352

6. **Albertsen M, Hugenholtz P, Skarshewski A, Nielsen KL, Tyson GW, Nielsen PH.** 2013. Genome sequences of rare, uncultured bacteria obtained by differential coverage binning of multiple metagenomes. Nature biotech 31:533-538. doi: 10.1038/nbt.2579

7. **Alneberg J, Bjarnason BS, de Bruijn I, Schirmer M, Quick J, Ijaz UZ, Lahti L, Loman NJ, Andersson AF, Quince C.** 2014. Binning metagenomic contigs by coverage and composition. Nat Meth 11:1144-1146. doi:10.1038/nmeth.3103

8. **Parks DH, Imelfort M, Skennerton CT, Hugenholtz P, Tyson GW.** 2015. CheckM: assessing the quality of microbial genomes recovered from isolates, single cells, and metagenomes. Genome Res doi:10.1101/gr.186072.114.

9. **Castelle CJ, Hug LA, Wrighton KC, Thomas BC, Williams KH, Wu D, Tringe SG, Singer SW, Eisen JA, Banfield JF.** 2013. Extraordinary phylogenetic diversity and metabolic versatility in aquifer sediment. Nat Commun 4. doi: 10.1038/ncomms3120

10. **Hug L, Baker BJ, Anantharaman K, Brown T, Probst AJ, astelle , Butterfield N, Hernsdorf AW,  Amano Y, Suzuki Y, Dudek N, Relman DA, Thomas B , Banfield JF** (2016) A new view of The Tree of  Life’s diversity. In review.

11. **Edgar RC.** 2004. MUSCLE: multiple sequence alignment with high accuracy and high throughput. Nucleic Acids Research 32:1792-1797.

12. **Capella-Gutiérrez S, Silla-Martínez JM, Gabaldón T.** 2009. trimAl: a tool for automated alignment trimming in large-scale phylogenetic analyses. Bioinformatics 25:1972-1973. doi: 10.1093/bioinformatics/btp348

13. **Darriba D, Taboada GL, Doallo R, Posada D.** 2011. ProtTest 3: fast selection of best-fit models of protein evolution. Bioinformatics 27: 1164-1165. doi: 10.1093/bioinformatics/btr088.

14. **Le SQ, Gascuel O.** 2008. An Improved General Amino Acid Replacement Matrix. Mol Biol Evol 25:1307-1320. doi: 10.1093/molbev/msn067

15. **Stamatakis A.** 2014. RAxML Version 8: A tool for Phylogenetic Analysis and Post-Analysis of Large Phylogenies. Bioinformatics 30: 1312-1313. doi: 10.1093/bioinformatics/btu033.

16. **Schloss P, Westcott S, Ryabin T, Hall J, Hartmann M, Hollister E, Lesniewski R, Oakley B, Parks D, Robinson C, Sahl J, Stres B, Thallinger G, Van Horn D, Weber C.** 2009. Introducing mothur: Open Source, Platform-independent, Community-supported Software for Describing and Comparing Microbial Communities. Appl Environ Microbiol doi:10.1128/AEM.01541-09.

17. **Pruesse E, Quast C, Knittel K, Fuchs BM, Ludwig W, Peplies J, Glöckner FO.** 2007. SILVA: a comprehensive online resource for quality checked and aligned ribosomal RNA sequence data compatible with ARB. Nucl Acid Res **35:**7188-7196. doi: 10.1093/nar/gkm864

18. **Ronquist F, Huelsenbeck JP.** 2003. MrBayes 3: Bayesian phylogenetic inference under mixed models. Bioinformatics **19:**1572-1574.

19. **Katoh K, Misawa K, Kuma K-i, Miyata T.** 2002. MAFFT: a novel method for rapid multiple sequence alignment based on fast Fourier transform. Nucleic Acids Research **30:**3059-3066.

20. **Dick G, Andersson A, Baker B, Simmons S, Thomas B, Yelton A, Banfield J.** 2009. Community-wide analysis of microbial genome sequence signatures. Genome Biol 10:R85. doi: 10.1186/gb-2009-10-8-r85.

21. **Daims H, Lebedeva EV, Pjevac P, Han P, Herbold C, Albertsen M, Jehmlich N, Palatinszky M, Vierheilig J, Bulaev A, Kirkegaard RH, Bergen Mv, Rattei T, Bendinger B, Nielsen PH, Wagner M.** 2015. Complete nitrification by Nitrospira bacteria. Nature **advance online publication**. doi: 10.1038/nature16461.

22. **van Kessel MAHJ, Speth DR, Albertsen M, Nielsen PH, Op den Camp HJM, Kartal B, Jetten MSM, Lücker S.** 2015. Complete nitrification by a single microorganism. Nature **advance online publication**. doi: 10.1038/nature16459.

23. **Hyatt D, Chen G-L, LoCascio P, Land M, Larimer F, Hauser L.** 2010. Prodigal: prokaryotic gene recognition and translation initiation site identification. BMC Bioinformatics **11:**119. Doi: 10.1186/1471-2105-11-119

24. **Zhao Y, Tang H, Ye Y.** 2012. RAPSearch2: a fast and memory-efficient protein similarity search tool for next-generation sequencing data. Bioinformatics **28:**125-126. Doi: 10.1093/bioinformatics/btr595
