# Supplementary figures and images for: Metagenomic Evidence for the Presence of Comammox Nitrospira-Like Bacteria in a Drinking Water System
Source: mSphere. 2015 Dec 30;1(1):e00054-15. doi: 10.1128/mSphere.00054-15 (PMC4863621; doi:10.1128/mSphere.00054-15)

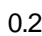

Supplement: Figure S1 [file sph001160049sf1.pdf]

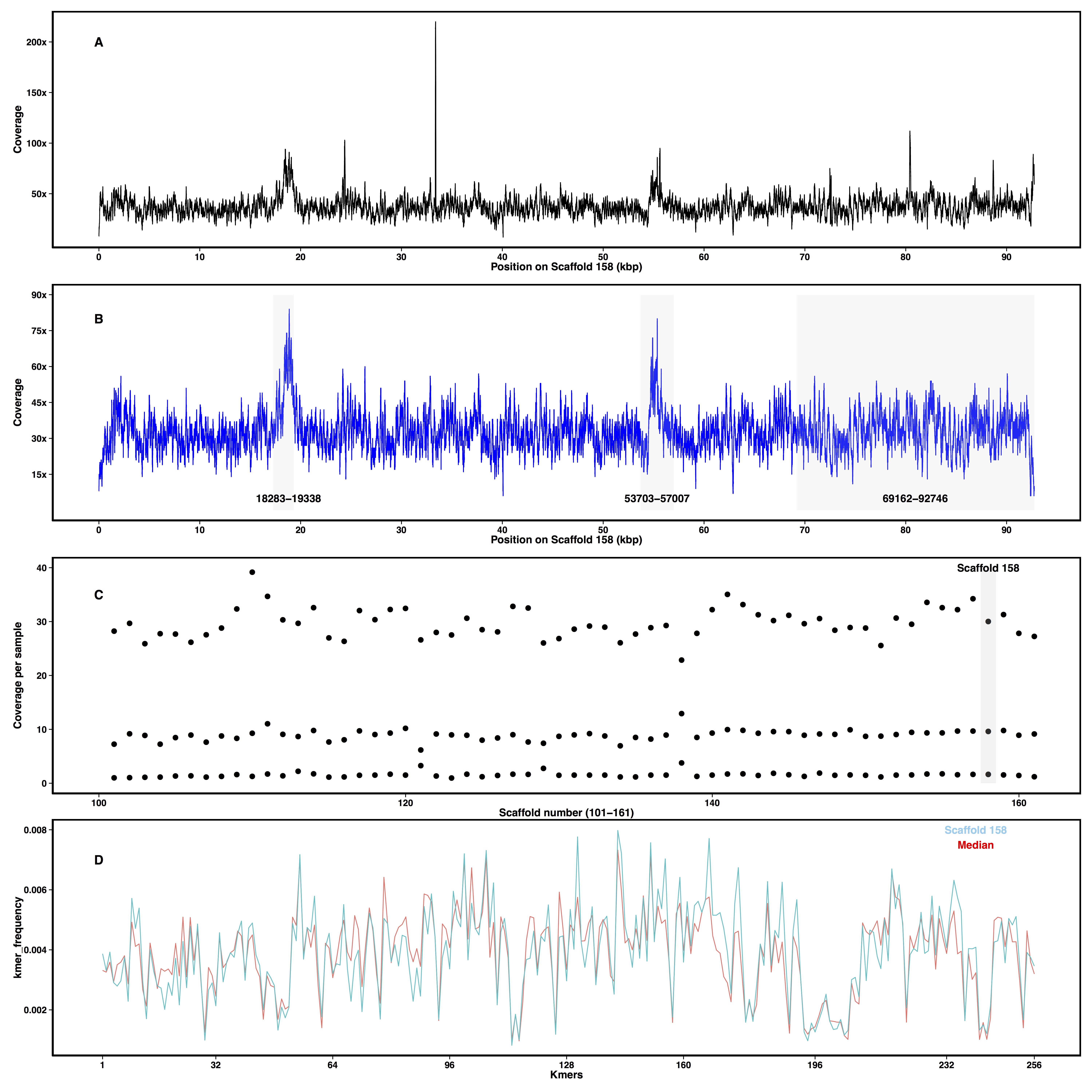

Supplement: Figure S2 [file sph001160049sf2.jpg]

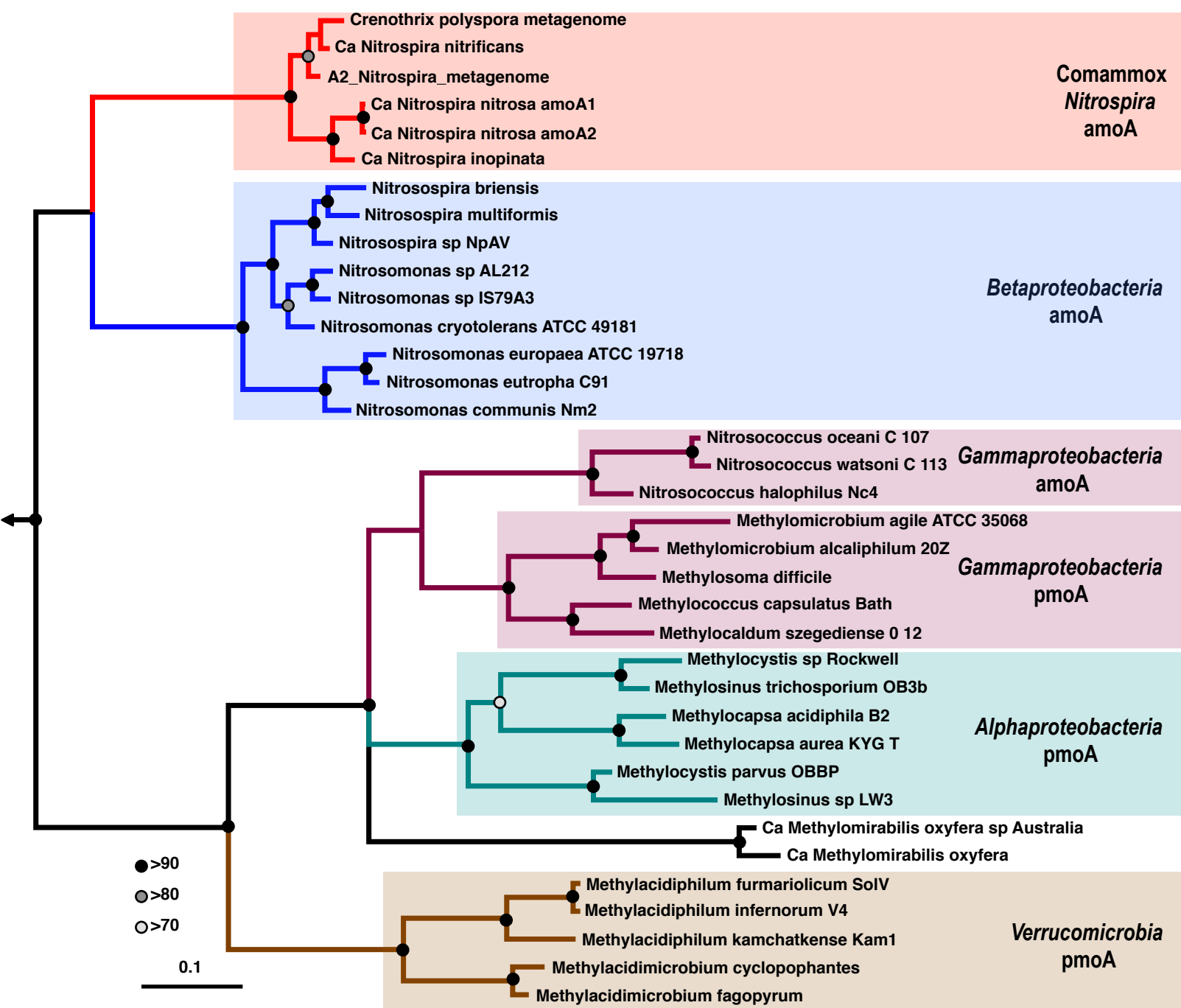

Supplement: Figure S3 [file sph001160049sf3.pdf]

A

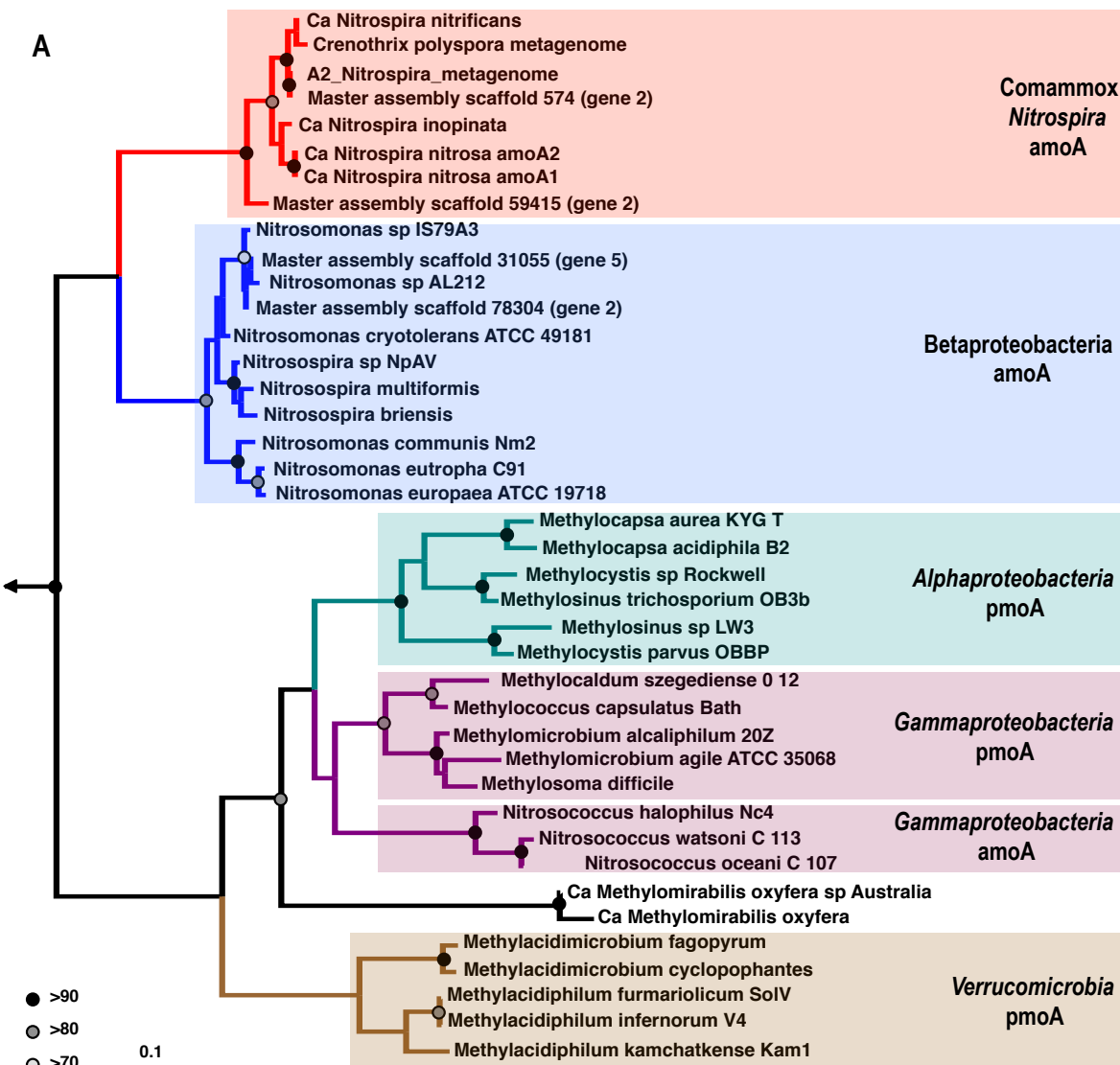

B

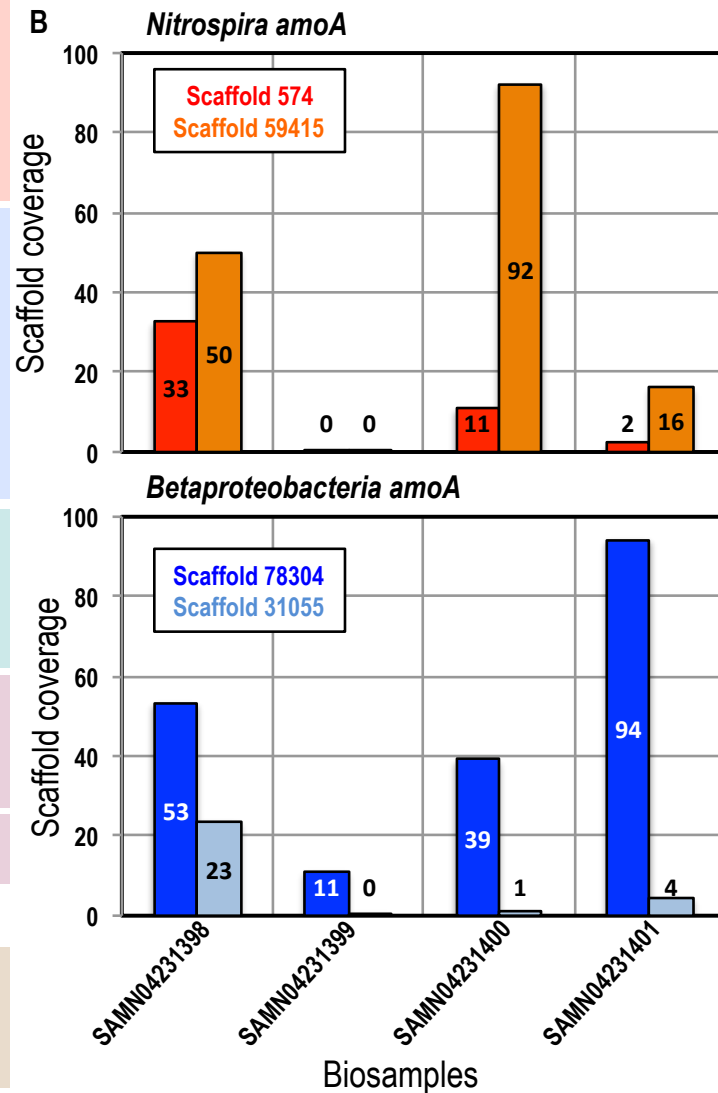

Supplement: Figure S4 [file sph001160049sf4.pdf]
